# The integrative bioinformatics approaches to predict the xanthohumol as anti-breast cancer molecule: Targeting cancer cells signaling PI3K and AKT kinase pathway

**DOI:** 10.3389/fonc.2022.950835

**Published:** 2022-12-15

**Authors:** Kartikey Kumar Gupta, Kamal Kant Sharma, Harish Chandra, Himalaya Panwar, Nitin Bhardwaj, Najla A. Altwaijry, Aisha A. Alsfouk, Zodwa Dlamini, Obaid Afzal, Abdulmalik S. A. Altamimi, Shahanavaj Khan, Abhay Prakash Mishra

**Affiliations:** ^1^ Department of Botany and Microbiology, Gurukula Kangri (Deemed to be University), Haridwar, Uttarakhand, India; ^2^ Department of Zoology and Environmental Science, Gurukula Kangri (Deemed to be University), Haridwar, Uttarakhand, India; ^3^ Department of Pharmaceutical Sciences, College of Pharmacy, Princess Nourah bint Abdulrahman University, Riyadh, Saudi Arabia; ^4^ SAMRC Precision Oncology Research Unit (PORU), DSI/NRF SARChI Chair in Precision Oncology and Cancer Prevention (POCP), Pan African Cancer Research Institute (PACRI), University of Pretoria, Hatfield, South Africa; ^5^ Department of Pharmaceutical Chemistry, College of Pharmacy, Prince Sattam bin Abdulaziz University, Al-Kharj, Saudi Arabia; ^6^ Department of Medical Lab Technology, Indian Institute of Health and Technology (IIHT), Saharanpur, Uttar Pradesh, India; ^7^ Department of Health Sciences, Novel Global Community Educational Foundation, Hebersham, NSW, Australia; ^8^ Department of Pharmaceutics, College of Pharmacy, King Saud University, King Saud University, Riyadh, Saudi Arabia; ^9^ Department of Pharmacology, University of Free State, Bloemfontein, Free State, South Africa

**Keywords:** breast cancer, *Humulus lupulus*, PI3K/AKT signaling, xanthohumol, anastrozole

## Abstract

**Background:**

Breast cancer is the most common type of cancer in women, and vast research is being conducted throughout the world for the treatment of this malignancy by natural products using various computational approaches. Xanthohumol, a prenylated flavonoid, is known for its anticancer activity; however, the mechanism behind its action is still in the preliminary stage.

**Methods:**

The current study aimed to analyze the efficacy of xanthohumol compared to the currently available anticancer drugs targeting phosphoinositide-3-kinase (PI3K), serine/threonine kinase (AKT) receptors, and human epidermal growth factor receptor 2 (HER2) for breast cancer treatment through *in silico* analysis.

**Results:**

The result revealed that the target compound showed significant binding affinity to targets within the PI3K, AKT, and HER2 signaling pathways with a binding energy of −7.5, −7.9, and −7.9 kcal/mol, respectively. Further prediction studies were then made concerning this compound’s absorption, distribution, metabolism, and excretion (ADME) as well as drug-likeness properties, resulting in its oral bioavailability with only a single violation of Lipinski’s rule of five.

**Conclusions:**

The finding revealed the ability of xanthohumol to bind with multiple cancer cell signaling molecules including PI3K, AKT kinase, and HER2. The current novel study opened the door to advancing research into the management and treatment of breast cancer.

## Introduction

Breast cancer is the second leading cause of death among women (1). Worldwide statistics in 2020 showed that out of all the types of cancers diagnosed, there are approximately 2.3 million newly diagnosed cases of breast cancer, accounting for about 11.7% of the total new cancer cases. This type of cancer is strictly related to age as a risk factor with only about 5% of cases confirmed in women less than 40 years of age (2). Currently, many therapeutic regimes have been developed to specifically inhibit key oncogenic targets, which play important roles in the growth of cancer. Consequently, in the inclusion or exclusion of chemotherapy, the administration of adjuvant tamoxifen reduces the possibility of death or tumor recurrence in women with hormone receptor-positive breast cancer (2, 3). This adjuvant treatment, which acts as an aromatase inhibitor for early-stage breast cancer, is prescribed for post-menopausal women diagnosed with hormone receptor- positive breast cancer (4–7). Clinically, different targeted therapies have also been reported to have fewer adverse effects in women suffering from various ailments including endometrial cancer, cerebrovascular complications, and thromboembolism (2, 3, 8).

According to previous studies, it has been observed that anastrozole considerably increased the period between tumor recurrence and illness-free survival (9, 10). Also, the administration of anastrozole is connected with less serious side effects than tamoxifen, such as fewer events of thrombo-embolism, ischemic cerebrovascular, and endometrial cancer but enhanced numbers of fractures following treatment (11). Additionally, several research studies on phosphoinositide-3-kinase (PI3K) and a serine/threonine-protein kinase, also known as AKT, signaling pathways have led to the development of numerous pathway inhibitors that are of important therapeutic use (12–14). PI3K/AKT signaling pathways have been recognized as the ideal pathway explaining the devolution of anti-apoptotic signals that promote cancer cell survival while also regulating cell growth, proliferation, transcription, and metabolic activities (14–16).

The natural moiety xanthohumol, a polyphenol chalcone from Hops (*Humulus lupulus*), has recently garnered interest due to its potent anticancer properties against colorectal, leukemia, lung, breast, and cervical cancer types, probably due to the presence of various phenolic compounds (17–19). Specifically, several investigations have shown that xanthohumol suppresses the growth of MCF-7 and SK-BR-3 breast cancer cell lines *in vivo* and *in vitro* experiments (20), but the exact mechanism through which xanthohumol mediates these effects is not clearly understood. Here, we attempt to predict the molecular mechanism of the anticancer activity of xanthohumol by analyzing the anticancer potential of xanthohumol through its ability to target the cell singling molecules human epidermal growth factor receptor 2 (HER2), PI3K, and AKT kinase using *in silico* approaches.

## Results

### Ramachandran and hydropathy plots

**Figure 1:**
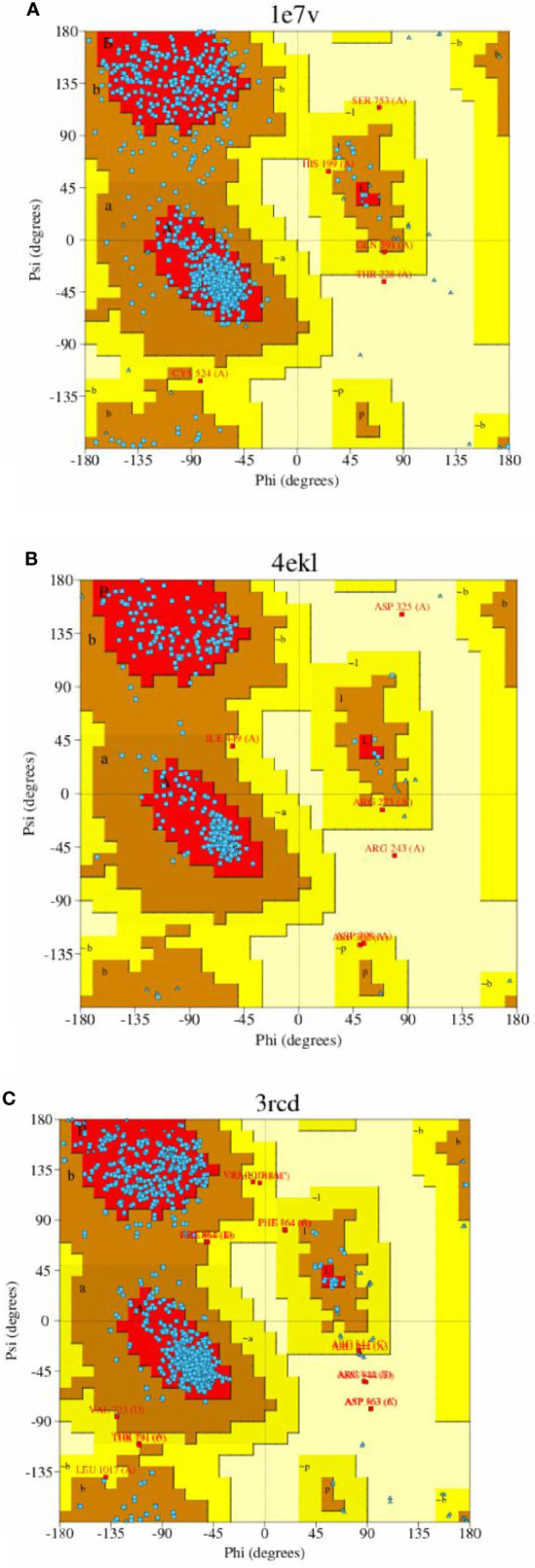
Stereo chemical quality of the target protein showing that the geometry of the protein target receptor corresponds to the highest likelihood conformation by torsional angles —phi and psi of the amino acid residues (Ramachandran plot) of target protein 1e7v (PIK) **(A)**, 4ekl (AKT) **(B)**, and 3rcd (HER2) **(C)**.

**Figure 2:**
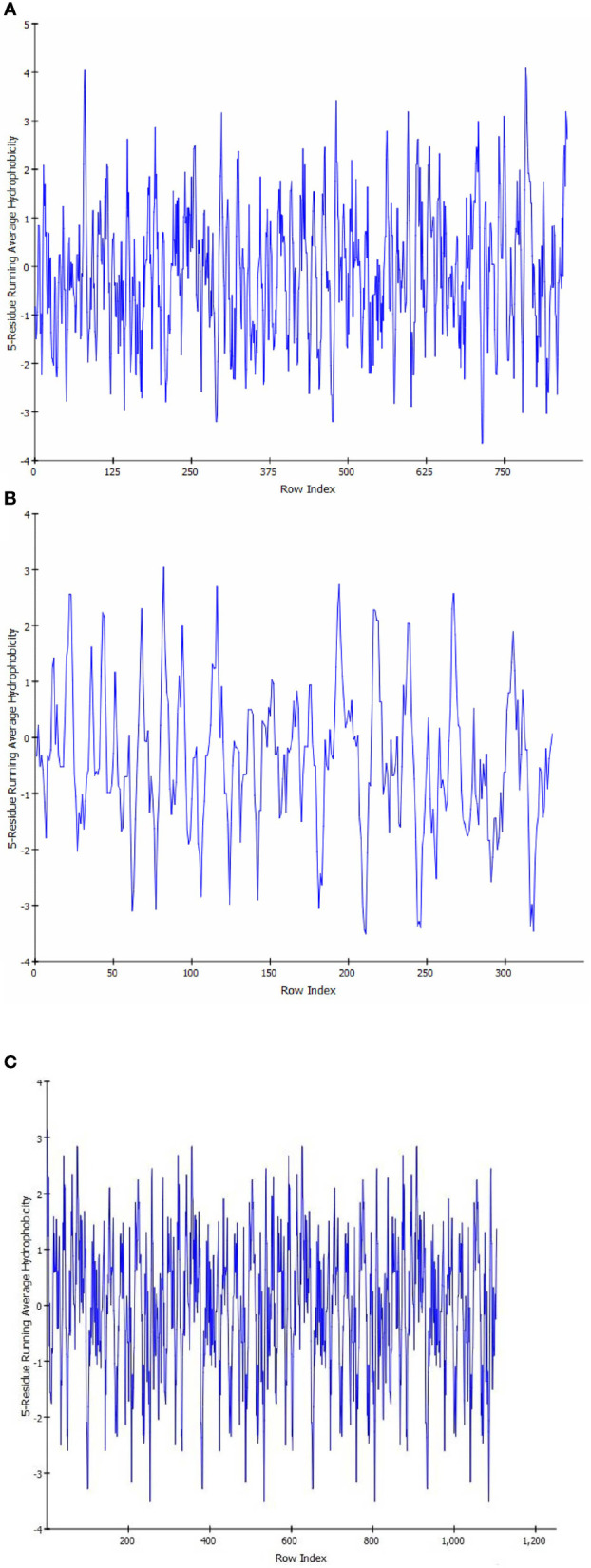
Hydropathy analysis showing the inclusion of hydrophilic and hydrophobic sections in the amino acid chain, beginning at the N terminal and progressing to the C terminal in target protein PIK **(A)**, AKT **(B)**, and HER2 **(C)**.

Sequence analysis revealed that PI3K and AKT proteins consisted of 850 and 330 amino acid residues, respectively. The Ramachandran plot showed that the stereochemical quality of the target protein demonstrated that the geometry of the protein target receptor corresponds to the highest likelihood conformation of the residues in the most favorable regions of the Ramachandran plot (**Figure 1**). The stereochemical property rule, following all the possible dihedral, phi (ϕ), and psi (ψ) angle values, are shown in **Table 1**. Protein structures were further corroborated by the inclusion of hydrophilic and hydrophobic sections in the amino acid chain, beginning at the N terminal and progressing to the C terminal (**Figure 2**).

**Table 1:** Summary of results from Ramachandran plot of the models.

Target model	Region			
	Most favored	Additional allowed	Generously allowed	Disallowed
PIK(1e7v)	85.1%	14.2%	0.5%	0.1%
AKT(4ekl)	91.4%	6.5%	1.4%	0.7%
HER2(3rcd)	87%	11.3%	1.3%	6.4%

### Molecular docking assessment for targeting HER2 and PI3K/AKT receptor

**Figure 3:**
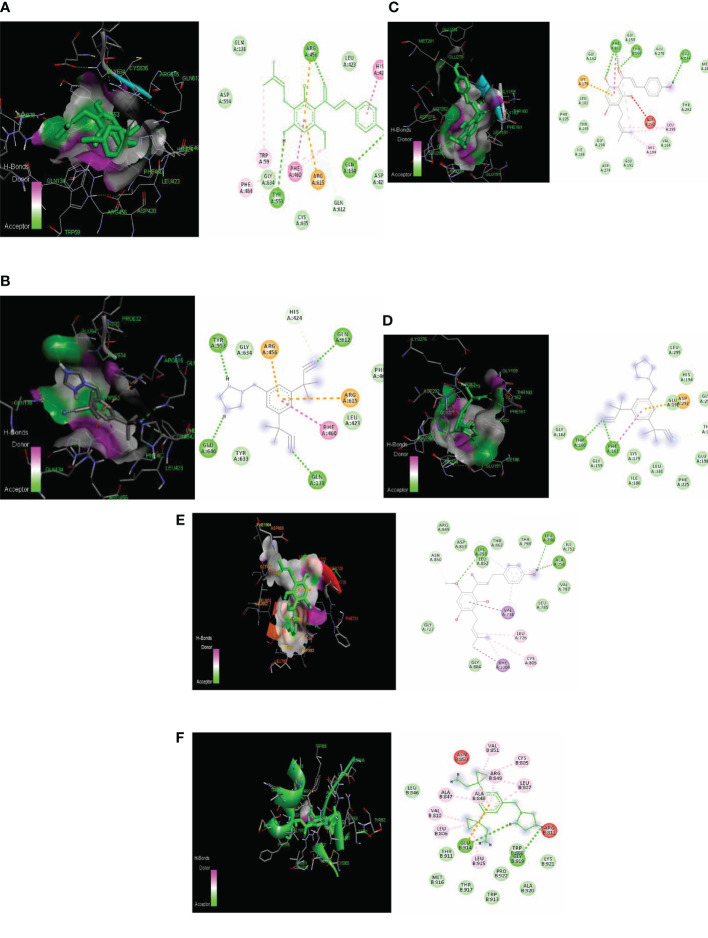
Lowest energy docking structure of **(A)** PI3K-Xanthohumol **(B)** PIK-Anastrozole, **(C)** AKT-Xanthohumol, **(D)** AKT-Anastrozole, **(E)** HER2-Xanthohumol, and **(F)** HER2-Anastrozole along with their 2D interactions.

The PI3K macromolecule interaction with xanthohumol targets the GLN 846, GLU 852, and LEU 845 residues with a binding energy of −7.5 kcal/mol (**Figure 3A**). Another receptor AKT possessed a binding site consisting of PHE 161, ASP 190, GLY 159, THR 160, and GLY 162 residues with a binding energy of −7.9 kcal/mol (**Figure 3B**). HER2 receptor interacts with xanthohumol at LYS 758, LEU 296, and ALA 751 residues with a binding energy of −7.9 kcal/mol (**Figure 3E**). However, the standard anastrozole interacted with PI3K at the active site consisting of ILE 881, VAL 882, LYS 883, ALA 885, and THR 886 amino acids with a binding energy of −7.7 kcal/mol (**Figure 3C**); AKT at residues LYS 189, GLU 191, ASP 190, ALA 193, and LEU 196 with a binding energy of −8.2 kcal/mol (**Figure 3D**); and HER2 receptor at GLU 914 and GLY 919 with a binding energy of −7.4 kcal/mol (**Figure 3F**).

Even though the tested ligand binds had similar energy, the plant- based derivative xanthohumol showed the same affinity to target the PI3K, AKT, and HER2 at active sites similar to those of the synthetic drug anastrozole (20) (**Table 2**).

**Table 2:** Representation of binding affinity and RMSD upper and lower bounds.

S. no.	Ligand	Binding Affinity (kcal/mol)	Inhibition constant (μM)
1.	PI3K-Xanthohumol	−7.5	3.14
2.	PI3K-Anastrozole	−7.5	3.14
3.	AKT-Xanthohumol	−7.9	1.59
4.	AKT-Anastrozole	−8.2	0.96
5.	HER2- Xanthohumol	−7.9	1.59
6.	HER2-Anastrozole	−7.4	3.39

RMSD, root mean square deviation.

### Absorption, distribution, metabolism, and excretion and drug-likeness properties

The drug-likeness and absorption, distribution, metabolism, and excretion (ADME) results of the xanthohumol are shown in **Table 3**. It was observed from the results that xanthohumol achieved the criteria of Lipinski’s rule of five, which meant it is likely a good drug, which is effective through oral administration. In a nutshell, these compounds are stated to have good absorptivity, less toxicity, oral bioavailability, and permeability.

**Table 3:** ADME and drug- likeness prediction.

Predictive model and their parameters	Compounds	
	Xanthohumol	Anastrozole
**Physicochemical properties**		
Molecular weight (g/mol)	354.40	293.37 g/mol
Fraction Csp3	0.19	0.41
Rotatable bond	6	4
H-bond acceptors	5	4
H-bond donors	3	0
Molar refractivity	102.53	83.81
TPSA^a^ (Å^2^)	86.99	78.29
**Lipophilicity**		
LogP_o/w_ ^b^ (XLOGP3)	5.07	2.03
LogP_o/w_ (WLOGP)	4.11	2.93
**Water solubility**		
Log S^c^ (ESOL)	−5.18	−3.04
Qualitative solubility	Moderately soluble	Soluble
**Drug-likeness**		
Lipinski (RO5)^d^	Yes; 0 violation	Yes; 0 violation
Ghose^e^	Yes	Yes
Veber^f^	Yes	Yes
Bioavailability score	0.55	0.55
**Lead-likeness**		
Rule of three (RO3)^g^	No; 2 violations: MW > 350	Yes
Synthetic accessibility^h^	3.16	2.21

a Topological polar surface area (TPSA).

b LogP_o/w_ is the partition coefficient between n-octanol and water.

c Log S is the decimal logarithm of the molar solubility in water.

d Lipinski (RO5) criteria range are lipophilicity (LogP_o/w_) ≤ 5, MW ≤ 500, H-bond ≤ 5, and H-bond acceptors ≤ 10.

e Ghose filter criteria range LogP_o/w_ in −0.4 to +5.6 range, MR from 40 to 130, MW from 180 to 480, and No. of atoms from 20 to 70.

f Veber’s rule criteria range are RB ≤ 10 and TPSA ≤ 140 Å^2^.

g RO3 criteria range are as follows: XLOGP3 ≤ 3.5, MW≤ 350, H-bond donors ≤3, H-bond acceptors ≤ 3, and Rotatable bond ≤ 3.

h Synthetic accessibility (SA) score ranges from 1 (very easy) to 10 (very difficult).

**Figure 4:**
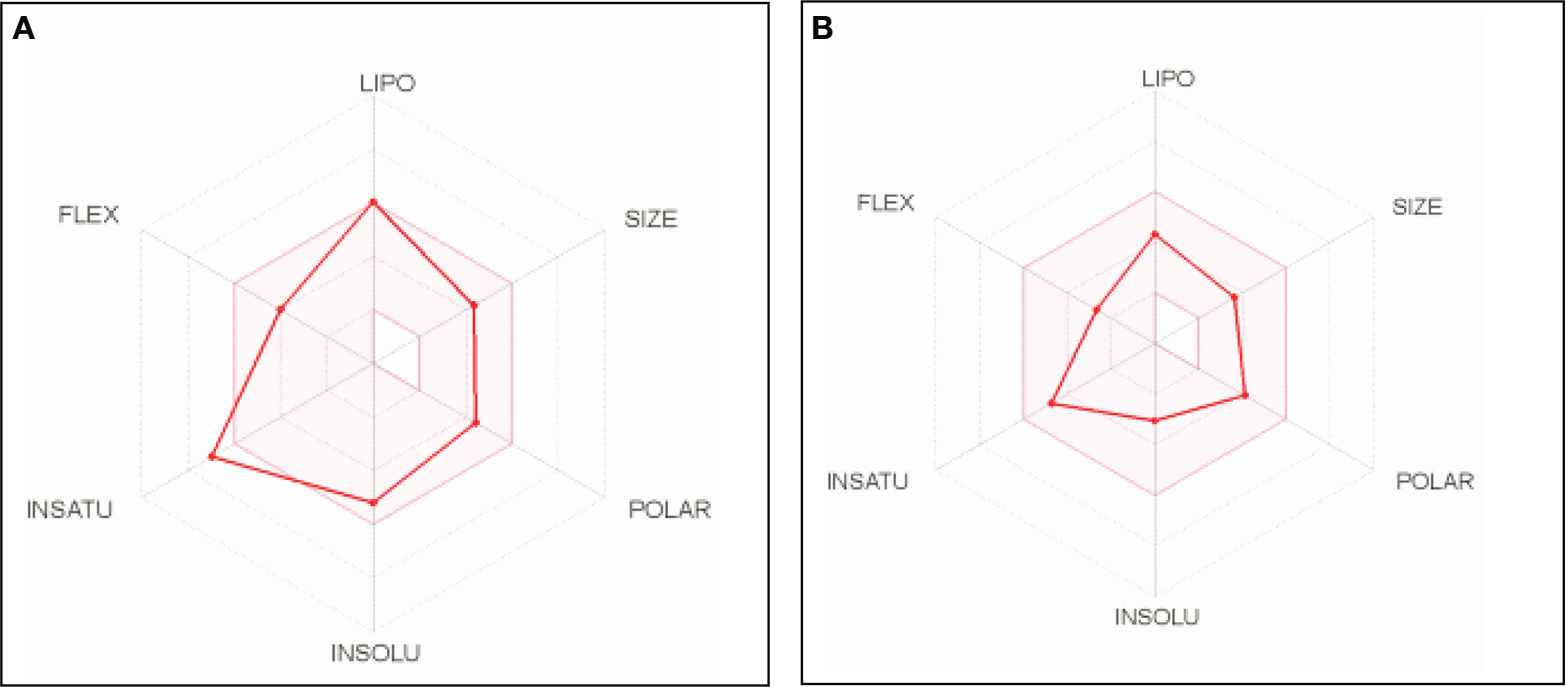
Bioavailability radar plots of **(A)** xanthohumol and **(B)** anastrozole. LIPO = lipophilicity (between −0.7 and +5.0), SIZE = molecular weight (between 150 and 500 g/mol), POLAR = polarity (between 20 and 130 Å^2^), INSOLU = solubility (not higher than 6), INSATU = saturation (fraction of carbons in the sp3 hybridization not less than 0.25), FLEX = flexibility (no more than 9 rotatable bonds).

The bioavailability radar plot provided a summary of a molecule’s drug-likeness (**Figure 4**). The effective range of each property of the molecule (xanthohumol and anastrozole) is indicated by the pink area (**Figures 4A, B**).

**Figure 5:**
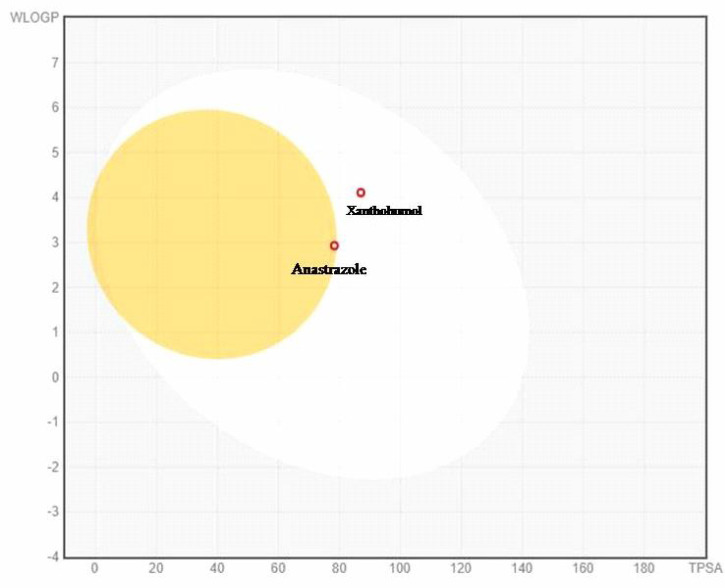
Boiled- egg plot between lipophilicity (WLOGP) and polarity (TPSA) showing anastrozole is able to permeate blood– brain barrier (BBB) and xanthohumol showing passive absorption by gastrointestinal tract. Molecules with red dots are anticipated to be unaffected by P-glycoprotein-mediated central nervous system (CNS) extrusion.

The Boiled-egg plot between WLOGP and topological polar surface area (TPSA) anticipates the gastrointestinal permeability and brain penetrating efficiency of the test molecules as shown in **Figure 5**. The plot showed that xanthohumol is rapidly absorbed in the gastrointestinal tract as compared to anastrozole, which permeates the blood– brain barrier by interaction with the inner yellow yolk. Furthermore, the PAINS (Pan-assay interference compounds) filter was used to evaluate the promiscuity of the primed hits. The results indicate that xanthohumol is a unique compound that bears no chemical resemblance to PAINS.

### Target prediction

**Figure 6:**
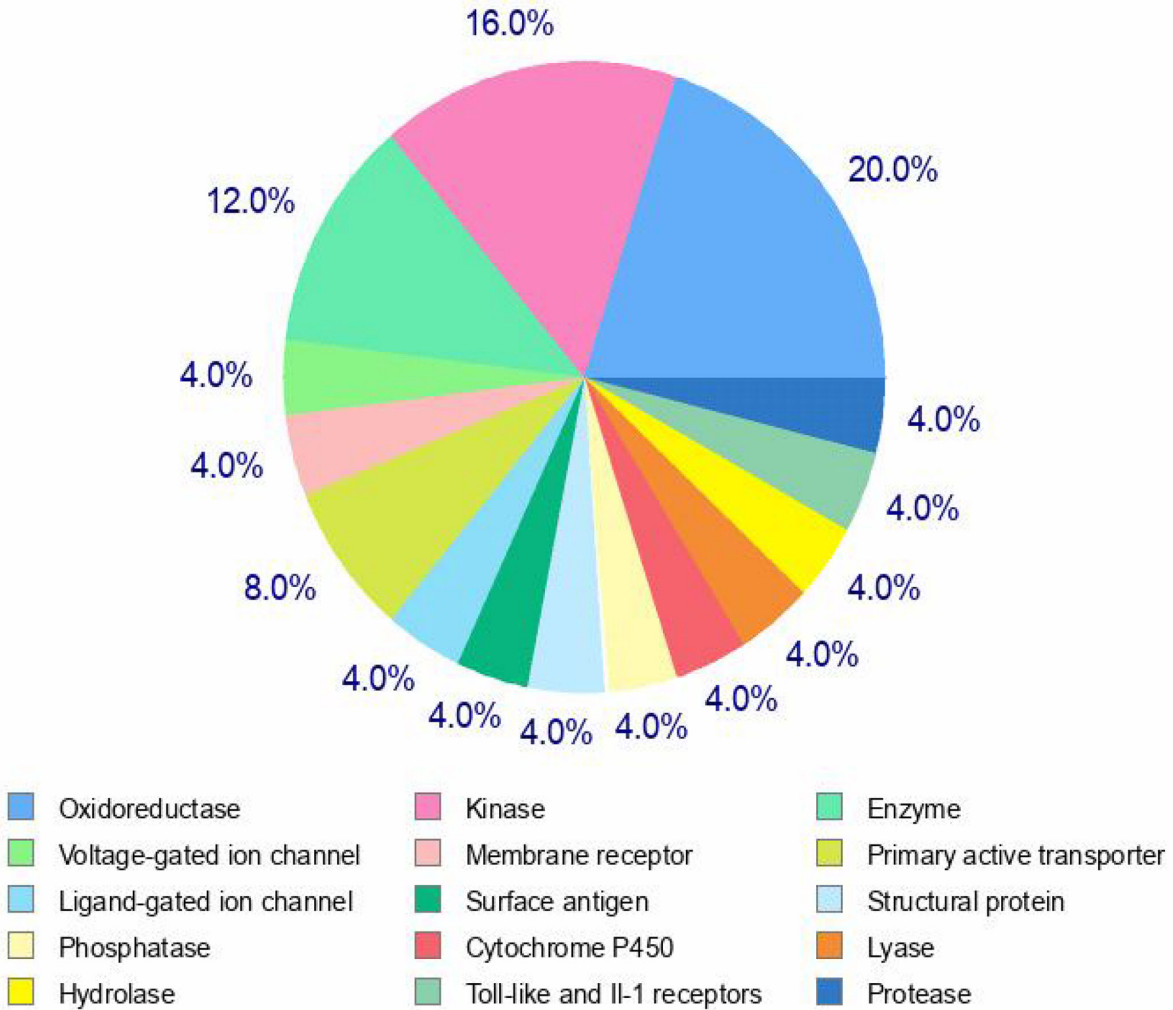
Top 25 targets predicted for xanthohumol.7.

A pie chart was developed using the top 25 results (probable targets) (**Figure 6**). The pie chart predicts that these top targets are made up of 20% of oxidoreductases, 16% of kinases, 12% of voltage- gated ion channels, 8% of primary active transporters, 4% of Toll-like and IL-1 receptors, 4% of cytochrome P450, 4% of hydrolases, and 4% of structural proteins. The various targets to which the chemical may bind are generally anticipated by the algorithms, and the likelihood score ranges from 0.376973 to 0.10934. This implies that the molecule may have a high affinity for the specific binding to which it is directed. The important conclusions were made as a result of the target predictions, which are shown on the server page (**Supplementary Table S1**).

### Toxicity prediction

The toxicity prediction was based on toxicity end points (Immunotoxicity, Carcinogenicity, Mutagenicity, and Cytotoxicity), organ toxicity (Hepatotoxicity), Tox21 Nuclear receptor signaling pathways, and Tox21 Stress response pathways. The results of toxicity prediction are summarized in **Table 4**. The LD_50_ of xanthohumol was found to be 3,800 mg/kg with class 5 predicted toxicity.

**Table 4:** Toxicity model report of xanthohumol.

Classification	Target	Shorthand	Result	Probability (%)
Organ toxicity	Hepatotoxicity	Dili	−	59
Toxicity end points	Carcinogenicity	Carcino	−	70
Toxicity end points	Immunotoxicity	Immune	+	99
Toxicity end points	Mutagenicity	Mutagen	−	72
Toxicity end points	Cytotoxicity	Cyto	−	69
Tox 21 —Nuclear receptor signaling pathways	Aryl hydrogen receptor (AhR)	nr_ahr	−	72
Tox 21 —Nuclear receptor signaling pathways	Androgen receptor (AR)	nr_ar	−	97
Tox 21— Nuclear receptor signaling pathways	Androgen receptor ligand binding domain (AR-LBD)	nr_ar_lbd	−	99
Tox 21— Nuclear receptor signaling pathways	Aromatase	nr_aromatase	−	80
Tox 21— Nuclear receptor signaling pathways	Estrogen receptor alpha	nr_er	+	51
Tox 21— Nuclear receptor signaling pathways	Estrogen receptor ligand binding domain (ER-LBD)	nr_er_lbd	−	65
Tox 21— Nuclear receptor signaling pathways	Peroxisome proliferator-activated receptor gamma (PPAR- Gamma)	nr_ppar_gamma	−	84
Tox 21— Stress response pathways	Nuclear factor (erythroid- derived 2)- like/antioxidant responsive element (Nrf2/ARE)	sr_are	−	56
Tox 21— Stress response pathways	Heat shock factor response element (HSE)	sr_hse	−	56
Tox 21— Stress response pathways	Mitochondrial membrane potential (MMP)	sr_mmp	+	81
Tox 21— Stress response pathways	Phosphoprotein (tumor suppressor) p53	sr_p53	−	52
Tox 21— Stress response pathways	ATPase family AAA domain-containing protein 5 (ATAD5)	sr_atad5	−	90

+, active; −, inactive.

## Discussion

*H. lupulus* (Hops), a primary raw ingredient in beer, has been widely employed in the brewing industry around the world. It serves as a preservative in the beer giving it characteristic scent and flavor (21, 22). Hops has also been implicated as a medicinal plant for a long time due to its high concentration of various phenolic compounds (23). Previously published reports have shown that dry hops consisted of about 4%–14% of polyphenolic compounds, flavonoids, catechins, prenylated chalcones, and proanthocyanidins (17). Although xanthohumol has demonstrated good chemopreventive activity in *in vitro* analysis, various other compounds (flavonoids, catechins, prenylated chalcones, and proanthocyanidins) of hops remain under preliminary research for the welfare of human beings. We also worked on other important compounds such as dehydrocycloxanthohumol and isoxanthohumol for the management of cancer through the best option of herbal medicine. We have planned to target the dehydrocycloxanthohumol and isoxanthohumol along with xanthohumol in our *in vivo* model study. The 8-prenylnaringenin has been characterized as one of the most potent phytoestrogens. Xanthohumol is a major prenylated flavonoid in hops and accounts for 0.1%–1% of the plants’ dry weight. The concentration of xanthohumol in beer has been observed to be up to 0.96 mg/L (1.95 μM) (17, 24, 25). It is renowned for its antioxidant, anti-inflammatory, antibacterial, antiviral, antifungal, anticancer, and anti-plasmodial properties (26, 27). Xanthohumol has gained much interest in recent years for its bioactivities, particularly in preventing and treating breast cancer. The related mechanisms underlying its anticancer properties have been revealed through the inhibition of carcinogenesis initiation and progression, as well as a therapeutic activity through proliferation suppression, induction of apoptosis, migration inhibition, and angiogenesis inhibition (28). Breast cancer is the most common cancer in women and the leading cause of cancer death globally (29). Cell propagation is a critical mechanism in carcinogenesis and disease progression (30). Changes in various cell signaling pathways increase cancer cell proliferation, growth, and survival. One such example is the PI3K/Akt/mTOR pathway (31), whose dysregulation has been linked to a wide range of cancer symptoms, including abnormal cell growth, genetic mutations, and metabolic reprogramming in cancerous cells; activation of this system is one of the main causes of cancer cell resistance to anticancer therapies (32). HER2 is a transmembrane receptor with intracellular tyrosine kinase activity. HER2 receptors usually aid in the regulation of normal breast cell development (33). However, amplification or overexpression of HER2 gene and its protein product have been observed in about 10% to 20% of breast cancers (34). Overexpression of this gene in the breast causes an increase in HER2 receptors leading to uncontrolled division and growth of breast cells. When a ligand binds to extracellular domains of HER proteins, it leads to dimerization and trans-phosphorylation of their intracellular domains, s ince HER2 receptors lack a ligand binding domain/site and can only be activated by homo-dimerization with itself and hetero-dimerization with another family member (35, 36). These phosphorylated tyrosine residues interact with a wide range of intracellular signaling molecules, resulting in secondary messenger activation and interaction with several other membrane signaling cascades (35). HER2 is considered a potent stimulator of the PI3K/AKT anti-apoptosis pathway (37, 38). A previous study has found that patients with estrogen receptor- positive/HER2- positive breast cancers may benefit more from drugs that disrupt the PI3K/AKT molecular pathway than patients with estrogen receptor- negative/HER2- positive breast cancers (37). Furthermore, activation of the PI3K/Akt pathway is one of the primary causes of the resistance of cancer cells to antitumor therapies (39). As a result, PI3K/Akt signaling is an important target for research into the genesis and course of cancer (31). The complex pathways that any chemical entity takes to reach its target frequently include the passage through many hurdles as well as the survival of the compound through a complex biological process (30). The bioavailability of any chemical entity is determined by a set of processes and several attributes, which can have a significant impact on its pharmacokinetic properties. In the past, the development of novel drugs had a significant attrition rate; approximately 40% of all treatment failures were connected to ADME difficulties (40). The combined assessment of the effectiveness and biological characteristics of active compounds has been standardized, and extensive investigations of ADME procedures are regularly performed at the preliminary phase of drug development to decrease the attrition rate. Computational approaches are being pursued by researchers to forecast the fate of a drug by determining the early risk of toxicity. *In silico* -based ADMET profiling approaches are frequently used to provide a basic idea before performing *in vitro* experiments.

Therefore, xanthohumol was used as a ligand to compare the compound’s potential with the reference drug (anastrozole) as an anticancer drug using molecular docking by utilizing the xanthohumol’s ability to bind and downregulate the PIP3/ATK pathway.

## Materials and methods

### Structure preparation

Crystallographic structures and sequences of PI3K, AKT, and HER2 were downloaded from the RCSB protein database (http://www.rcsb.org) with Protein Data Bank (PDB) ID 1E7V, 4EKL, and 3RCD respectively. Water and heteroatoms including inhibitors were manually removed from the receptor molecule. The structures of the ligands xanthohumol and anastrozole (reference drug) were made using RPBS frog 2 web portal servers *via* canonical SMILES obtained from the Pubchem database with Pubchem IDs of 639665 and 2187, respectively (32, 41) (**Table 5**). The Mol2 file format of the ligands was downloaded from the RPBS frog server, which was then converted into PDB file format through Open Babel 2.4.1 software (23). The chimera UCSF suite was used to eliminate the steric clashes on the targets (PI3K and AKT) and ligand molecules by energy minimization steps after the addition of H-atoms (42).

**Table 5:** PubChem ID and canonical SMILES of xanthohumol and anastrozole.

Ligands	PubC hem ID	Canonical SMILES
Xanthohumol	639665	CC(=CCC1=C(C(=C(C=C1O)OC)C(=O)C=CC2=CC=C(C=C2)O)O)C
Anastrozole	2187	CC(C)(C#N)C1=CC(=CC(=C1)CN2C=NC=N2)C(C)(C)C#N

### Ramachandran plot and hydropathy analysis

The stereochemical quality of the protein structure retrieved from PDB was checked using the Procheck server (43). Ramachandran plot analysis was performed to test for the significance of the position of the secondary structural features, such as alpha helices and beta sheets, and the polypeptide backbone was decided by Ø & Ψ bond angles in the protein conformation. The angles depicted in the Ramachandran plot were employed for protein modeling and determination of structural properties. The statistical parameters of the protein exhibited the distribution of amino acids in the secondary structure (44). The hydropathy plot was analyzed using BIOVIA Software (Discovery Studio) for the validation of the hydrophilic or hydrophobic nature of the receptor molecule.

### Molecular docking

In order to assess the efficacy of xanthohumol and anastrozole for targeting PI3K and AKT proteins, a molecular docking study was carried out at their respective catalytic targets. The input contained flexible receptor, rigid receptor, docking box, and ligand, while the output involved a catalog of models ranked by the predicted binding energy in kcal/mol. The target protein and selected ligand compounds were primed *via* the PyRx software (45). Docking simulations of PI3K, AKT, and HER2 were initiated with active sites as predicted by the CASTp server (46). The docking model with the lowest energy was extracted and aligned with the receptor using the Discovery Studio Visualizer. The binding efficacy of ligands to receptors was calculated according to the Lamarckian genetic algorithm (LGA) with a maximum of 250,000 (units) energy. Following this, the inhibition constant (Ki) was calculated from the binding energy (ΔG) using the formula Ki = exp (ΔG/RT), where R is acting as the universal gas constant (1.985 × 10^−3^ kcal mol^−1^ K^−1^) and T is the temperature (298.15 K).

### Absorption, distribution, metabolism, and excretion properties and drug-likeness prediction

The absorption, distribution, metabolism, and excretion (ADME) properties and drug-likeness prediction of ligands were analyzed using Swiss ADME a freely available web tool (47). Lipinski’s rule of five criteria were used for the determination of the drug-likeness of the molecule. That is, the number of hydrogen bond donors is 5, and the number of hydrogen bond acceptors is 10. The molecular weight of the drug is < 500 Da. A calculated Log p ≤ 5 and polar surface area (PSA) ≤ 140 Å^2^. Violation of more than two of these criteria indicates that the test molecule is said to be impermeable or not orally bioavailable (48).

### Target prediction

Pharmacological investigations are critical for identifying possible cross-reactivity or phenotypic side effects induced by the interaction of small biomolecules (28). For target prediction studies, canonical SMILES of xanthohumol were put in the Swiss Target Prediction webserver, and outputs were analyzed.

### Toxicity prediction

The prediction of a molecule’s toxicological profile is critical for estimating the molecule’s acceptability before it is administered in both animal and human models. The toxicity of the molecule was analyzed by the Protox II server (https://toxnew.charite.de/protox_II/index.php?site=compound_input). The canonical SMILES of xanthohumol retrieved from PubChem were used as input to predict toxicity (49).

## Conclusions

Through this *in silico* docking study, we examined the potency of the binding capacity of xanthohumol against breast cancer cell signaling molecules, namely, PI3K and AKT, which are reported to be involved in proliferation, regulating cell growth, transcription, and metabolic processes. A comparative docking study of xanthohumol with a commercially available drug anastrozole revealed that the binding affinity of xanthohumol is comparable or in some cases even better than the specific signaling inhibitor function of anastrozole- like drugs, which are already in clinical trials. Therefore, xanthohumol appears to be an alternative candidate to the presently available anastrozole- like drugs. However, further *in vitro* and *in vivo* studies followed by stepwise clinical trials are required to establish it as an effective drug candidate.

## Data availability statement

The original contributions presented in the study are included in the article/**Supplementary Material**. Further inquiries can be directed to the corresponding authors.

## Author contributions

Idea and concept: KG and KS. Data analysis, prediction work, and original draft preparation: HC, HP, NB, and ZD Critical review and editing work: OA and ASAA Editing work, supervision, and funding acquisition: NA, AAA, AM, and SK. All authors have read and agreed to the published version of the manuscript.
