# Supplementary material for: The integrative bioinformatics approaches to predict the xanthohumol as anti-breast cancer molecule: Targeting cancer cells signaling PI3K and AKT kinase pathway
Source: Front Oncol. 2022 Dec 15;12:950835. doi: 10.3389/fonc.2022.950835 (PMC9798915; doi:10.3389/fonc.2022.950835)
Supplement: Supplementary file 1 [file Table_1.doc]

**Supplementary Table S1:** The various targets to which the chemical may bind are generally anticipated by the algorithms with likelihood score ranges from 0.376973 to 0.10934.

| **Target** | **Common name** | **Uniprot ID** | **ChEMBL ID** | **Target Class** | **Probability*** | **Known actives (3D/2D)** |
| --- | --- | --- | --- | --- | --- | --- |
| Arachidonate 5-lipoxygenase | ALOX5 | P09917 | CHEMBL215 | Oxidoreductase | 0.376973 | 0 / 42Â Â Â Â Â |
| 3-phosphoinositide dependent protein kinase-1 | PDPK1 | O15530 | CHEMBL2534 | Kinase | 0.376973 | 0 / 1Â Â Â Â Â |
| Aldose reductase (by homology) | AKR1B1 | P15121 | CHEMBL1900 | Enzyme | 0.149917 | 3 / 40Â Â Â Â Â |
| Voltage-gated potassium channel subunit Kv1.3 | KCNA3 | P22001 | CHEMBL4633 | Voltage-gated ion channel | 0.149917 | 0 / 9Â Â Â Â Â |
| Beta amyloid A4 protein | APP | P05067 | CHEMBL2487 | Membrane receptor | 0.133684 | 0 / 15Â Â Â Â Â |
| Epidermal growth factor receptor erbB1 | EGFR | P00533 | CHEMBL203 | Kinase | 0.117455 | 5 / 15Â Â Â Â Â |
| ATP-binding cassette sub-family G member 2 | ABCG2 | Q9UNQ0 | CHEMBL5393 | Primary active transporter | 0.10934 | 1 / 54Â Â Â Â Â |
| Neuronal acetylcholine receptor protein alpha-7 subunit | CHRNA7 | P36544 | CHEMBL2492 | Ligand-gated ion channel | 0.10934 | 0 / 11Â Â Â Â Â |
| Coagulation factor VII/tissue factor | F3 | P13726 | CHEMBL4081 | Surface antigen | 0.10934 | 0 / 6Â Â Â Â Â |
| Tubulin beta-1 chain | TUBB1 | Q9H4B7 | CHEMBL1915 | Structural protein | 0.10934 | 0 / 16Â Â Â Â Â |
| Monoamine oxidase B | MAOB | P27338 | CHEMBL2039 | Oxidoreductase | 0.10934 | 0 / 114Â Â Â Â Â |
| Protein-tyrosine phosphatase 1B | PTPN1 | P18031 | CHEMBL335 | Phosphatase | 0.10934 | 6 / 34Â Â Â Â Â |
| Monoamine oxidase A | MAOA | P21397 | CHEMBL1951 | Oxidoreductase | 0.10934 | 0 / 31Â Â Â Â Â |
| Cytochrome P450 19A1 | CYP19A1 | P11511 | CHEMBL1978 | Cytochrome P450 | 0.10934 | 2 / 39Â Â Â Â Â |
| Nitric oxide synthase, inducible (by homology) | NOS2 | P35228 | CHEMBL4481 | Enzyme | 0.10934 | 2 / 10Â Â Â Â Â |
| Ornithine decarboxylase | ODC1 | P11926 | CHEMBL1869 | Lyase | 0.10934 | 0 / 6Â Â Â Â Â |
| Telomerase reverse transcriptase | TERT | O14746 | CHEMBL2916 | Enzyme | 0.10934 | 0 / 2Â Â Â Â Â |
| MAP kinase-activated protein kinase 2 | MAPKAPK2 | P49137 | CHEMBL2208 | Kinase | 0.10934 | 0 / 2Â Â Â Â Â |
| MAP kinase-activated protein kinase 5 | MAPKAPK5 | Q8IW41 | CHEMBL3094 | Kinase | 0.10934 | 0 / 1Â Â Â Â Â |
| Aldehyde dehydrogenase | ALDH2 | P05091 | CHEMBL1935 | Oxidoreductase | 0.10934 | 0 / 1Â Â Â Â Â |
| P-glycoprotein 1 | ABCB1 | P08183 | CHEMBL4302 | Primary active transporter | 0.10934 | 7 / 20Â Â Â Â Â |
| Acetylcholinesterase | ACHE | P22303 | CHEMBL220 | Hydrolase | 0.10934 | 0 / 72Â Â Â Â Â |
| Cyclooxygenase-1 | PTGS1 | P23219 | CHEMBL221 | Oxidoreductase | 0.10934 | 1 / 13Â Â Â Â Â |
| Toll-like receptor (TLR7/TLR9) | TLR9 | Q9NR96 | CHEMBL5804 | Toll-like and Il-1 receptors | 0.10934 | 1 / 6Â Â Â Â Â |
| Thrombin and coagulation factor X | F10 | P00742 | CHEMBL244 | Protease | 0.10934 | 22 / 0Â Â Â Â Â |
| Cyclooxygenase-2 | PTGS2 | P35354 | CHEMBL230 | Oxidoreductase | 0.10934 | 1 / 13Â Â Â Â Â |
| Cysteinyl leukotriene receptor 2 | CYSLTR2 | Q9NS75 | CHEMBL4330 | Family A G protein-coupled receptor | 0.10934 | 0 / 2Â Â Â Â Â |
| Stem cell growth factor receptor | KIT | P10721 | CHEMBL1936 | Kinase | 0.10934 | 0 / 2Â Â Â Â Â |
| Peroxisome proliferator-activated receptor gamma | PPARG | P37231 | CHEMBL235 | Nuclear receptor | 0.10934 | 0 / 18Â Â Â Â Â |
| Protein kinase C delta | PRKCD | Q05655 | CHEMBL2996 | Kinase | 0.10934 | 28 / 1Â Â Â Â Â |
| Beta-secretase 1 | BACE1 | P56817 | CHEMBL4822 | Protease | 0.10934 | 3 / 22Â Â Â Â Â |
| Induced myeloid leukemia cell differentiation protein Mcl-1 | MCL1 | Q07820 | CHEMBL4361 | Other cytosolic protein | 0 | 6 / 7Â Â Â Â Â |
| Alpha-synuclein | SNCA | P37840 | CHEMBL6152 | Unclassified protein | 0 | 0 / 3Â Â Â Â Â |
| ALK tyrosine kinase receptor | ALK | Q9UM73 | CHEMBL4247 | Kinase | 0 | 1 / 0Â Â Â Â Â |
| Nuclear factor NF-kappa-B p65 subunit | RELA | Q04206 | CHEMBL5533 | Transcription factor | 0 | 1 / 7Â Â Â Â Â |
| Urokinase-type plasminogen activator | PLAU | P00749 | CHEMBL3286 | Protease | 0 | 6 / 0Â Â Â Â Â |
| Endoplasmin | HSP90B1 | P14625 | CHEMBL1075323 | Other membrane protein | 0 | 40 / 0Â Â Â Â Â |
| Aldo-keto-reductase family 1 member C3 | AKR1C3 | P42330 | CHEMBL4681 | Enzyme | 0 | 0 / 2Â Â Â Â Â |
| C-X-C chemokine receptor type 4 | CXCR4 | P61073 | CHEMBL2107 | Family A G protein-coupled receptor | 0 | 0 / 1Â Â Â Â Â |
| Interleukin-8 receptor B | CXCR2 | P25025 | CHEMBL2434 | Family A G protein-coupled receptor | 0 | 28 / 0Â Â Â Â Â |
| Butyrylcholinesterase | BCHE | P06276 | CHEMBL1914 | Hydrolase | 0 | 0 / 8Â Â Â Â Â |
| Adenosine A2b receptor | ADORA2B | P29275 | CHEMBL255 | Family A G protein-coupled receptor | 0 | 1 / 0Â Â Â Â Â |
| Rho-associated protein kinase 2 | ROCK2 | O75116 | CHEMBL2973 | Kinase | 0 | 1 / 0Â Â Â Â Â |
| Arachidonate 12-lipoxygenase | ALOX12 | P18054 | CHEMBL3687 | Enzyme | 0 | 2 / 0Â Â Â Â Â |
| Histamine H3 receptor | HRH3 | Q9Y5N1 | CHEMBL264 | Family A G protein-coupled receptor | 0 | 0 / 1Â Â Â Â Â |
| Prostanoid EP4 receptor | PTGER4 | P35408 | CHEMBL1836 | Family A G protein-coupled receptor | 0 | 0 / 11Â Â Â Â Â |
| Prostanoid EP2 receptor | PTGER2 | P43116 | CHEMBL1881 | Family A G protein-coupled receptor | 0 | 0 / 12Â Â Â Â Â |
| Prostanoid EP3 receptor | PTGER3 | P43115 | CHEMBL3710 | Family A G protein-coupled receptor | 0 | 0 / 11Â Â Â Â Â |
| Estradiol 17-beta-dehydrogenase 2 | HSD17B2 | P37059 | CHEMBL2789 | Enzyme | 0 | 2 / 0Â Â Â Â Â |
| Estradiol 17-beta-dehydrogenase 1 | HSD17B1 | P14061 | CHEMBL3181 | Enzyme | 0 | 2 / 0Â Â Â Â Â |
| Dihydroorotate dehydrogenase | DHODH | Q02127 | CHEMBL1966 | Oxidoreductase | 0 | 0 / 1Â Â Â Â Â |
| Protein kinase C alpha | PRKCA | P17252 | CHEMBL299 | Kinase | 0 | 29 / 0Â Â Â Â Â |
| Protein kinase C beta | PRKCB | P05771 | CHEMBL3045 | Kinase | 0 | 32 / 0Â Â Â Â Â |
| Protein kinase C epsilon | PRKCE | Q02156 | CHEMBL3582 | Kinase | 0 | 28 / 0Â Â Â Â Â |
| C-C chemokine receptor type 4 | CCR4 | P51679 | CHEMBL2414 | Family A G protein-coupled receptor | 0 | 7 / 0Â Â Â Â Â |
| Proteasome assembly chaperone 3 | PSMG3 | Q9BT73 | CHEMBL1075137 | Unclassified protein | 0 | 1 / 0Â Â Â Â Â |
| Prostaglandin E synthase | PTGES | O14684 | CHEMBL5658 | Enzyme | 0 | 4 / 26Â Â Â Â Â |
| Cyclin-dependent kinase 5/CDK5 activator 1 | CDK5R1 CDK5 | Q15078 Q00535 | CHEMBL1907600 | Kinase | 0 | 0 / 1Â Â Â Â Â |
| Plasminogen activator inhibitor-1 | SERPINE1 | P05121 | CHEMBL3475 | Secreted protein | 0 | 1 / 0Â Â Â Â Â |
| Interleukin-8 receptor A | CXCR1 | P25024 | CHEMBL4029 | Family A G protein-coupled receptor | 0 | 1 / 0Â Â Â Â Â |
| Endothelin receptor ET-A (by homology) | EDNRA | P25101 | CHEMBL252 | Family A G protein-coupled receptor | 0 | 0 / 32Â Â Â Â Â |
| Inosine-5'-monophosphate dehydrogenase 2 | IMPDH2 | P12268 | CHEMBL2002 | Oxidoreductase | 0 | 0 / 22Â Â Â Â Â |
| Cyclin-dependent kinase 4/cyclin D1 | CCND1 CDK4 | P24385 P11802 | CHEMBL1907601 | Kinase | 0 | 2 / 0Â Â Â Â Â |
| Protein kinase C gamma | PRKCG | P05129 | CHEMBL2938 | Kinase | 0 | 26 / 0Â Â Â Â Â |
| Protein kinase C eta | PRKCH | P24723 | CHEMBL3616 | Kinase | 0 | 27 / 0Â Â Â Â Â |
| Leukocyte elastase | ELANE | P08246 | CHEMBL248 | Protease | 0 | 0 / 17Â Â Â Â Â |
| Retinoic acid receptor gamma | RARG | P13631 | CHEMBL2003 | Nuclear receptor | 0 | 0 / 15Â Â Â Â Â |
| Retinoic acid receptor beta | RARB | P10826 | CHEMBL2008 | Nuclear receptor | 0 | 0 / 13Â Â Â Â Â |
| Retinoic acid receptor alpha | RARA | P10276 | CHEMBL2055 | Nuclear receptor | 0 | 0 / 12Â Â Â Â Â |
| Vascular endothelial growth factor receptor 1 | FLT1 | P17948 | CHEMBL1868 | Kinase | 0 | 0 / 1Â Â Â Â Â |
| Histone deacetylase 2 | HDAC2 | Q92769 | CHEMBL1937 | Eraser | 0 | 0 / 1Â Â Â Â Â |
| Platelet-derived growth factor receptor alpha | PDGFRA | P16234 | CHEMBL2007 | Kinase | 0 | 0 / 1Â Â Â Â Â |
| Serine/threonine-protein kinase Aurora-C | AURKC | Q9UQB9 | CHEMBL3935 | Kinase | 0 | 0 / 1Â Â Â Â Â |
| Cathepsin L | CTSL | P07711 | CHEMBL3837 | Protease | 0 | 0 / 4Â Â Â Â Â |
| Squalene monooxygenase (by homology) | SQLE | Q14534 | CHEMBL3592 | Enzyme | 0 | 1 / 0Â Â Â Â Â |
| Apoptosis regulator Bcl-X | BCL2L1 | Q07817 | CHEMBL4625 | Other ion channel | 0 | 1 / 0Â Â Â Â Â |
| cAMP-dependent protein kinase alpha-catalytic subunit | PRKACA | P17612 | CHEMBL4101 | Kinase | 0 | 10 / 0Â Â Â Â Â |
| Egl nine homolog 1 | EGLN1 | Q9GZT9 | CHEMBL5697 | Oxidoreductase | 0 | 49 / 0Â Â Â Â Â |
| Glycogen synthase kinase-3 beta | GSK3B | P49841 | CHEMBL262 | Kinase | 0 | 1 / 3Â Â Â Â Â |
| Receptor protein-tyrosine kinase erbB-2 | ERBB2 | P04626 | CHEMBL1824 | Kinase | 0 | 5 / 2Â Â Â Â Â |
| C-C chemokine receptor type 5 | CCR5 | P51681 | CHEMBL274 | Family A G protein-coupled receptor | 0 | 1 / 0Â Â Â Â Â |
| Poly [ADP-ribose] polymerase-1 | PARP1 | P09874 | CHEMBL3105 | Enzyme | 0 | 0 / 15Â Â Â Â Â |
| Phosphodiesterase 5A | PDE5A | O76074 | CHEMBL1827 | Phosphodiesterase | 0 | 2 / 0Â Â Â Â Â |
| Steryl-sulfatase | STS | P08842 | CHEMBL3559 | Enzyme | 0 | 0 / 2Â Â Â Â Â |
| Isocitrate dehydrogenase [NADP] cytoplasmic | IDH1 | O75874 | CHEMBL2007625 | Enzyme | 0 | 1 / 0Â Â Â Â Â |
| Thrombin | F2 | P00734 | CHEMBL204 | Protease | 0 | 8 / 0Â Â Â Â Â |
| Fatty acid synthase | FASN | P49327 | CHEMBL4158 | Transferase | 0 | 1 / 0Â Â Â Â Â |
| Dual specificity protein phosphatase 3 | DUSP3 | P51452 | CHEMBL2635 | Phosphatase | 0 | 2 / 0Â Â Â Â Â |
| Microtubule-associated protein tau | MAPT | P10636 | CHEMBL1293224 | Unclassified protein | 0 | 0 / 1Â Â Â Â Â |
| G-protein coupled receptor 84 | GPR84 | Q9NQS5 | CHEMBL3714079 | Family A G protein-coupled receptor | 0 | 1 / 0Â Â Â Â Â |
| Tyrosine-protein kinase LCK | LCK | P06239 | CHEMBL258 | Kinase | 0 | 0 / 15Â Â Â Â Â |
| DNA excision repair protein ERCC-5 | ERCC5 | P28715 | CHEMBL4736 | Other nuclear protein | 0 | 2 / 0Â Â Â Â Â |
| Pyruvate dehydrogenase kinase isoform 1 | PDK1 | Q15118 | CHEMBL4766 | Kinase | 0 | 6 / 0Â Â Â Â Â |
| Flap endonuclease 1 | FEN1 | P39748 | CHEMBL5027 | Enzyme | 0 | 4 / 0Â Â Â Â Â |
| Peroxisome proliferator-activated receptor alpha | PPARA | Q07869 | CHEMBL239 | Nuclear receptor | 0 | 0 / 9Â Â Â Â Â |
| Peroxisome proliferator-activated receptor delta | PPARD | Q03181 | CHEMBL3979 | Nuclear receptor | 0 | 0 / 4Â Â Â Â Â |
| ADAMTS5 | ADAMTS5 | Q9UNA0 | CHEMBL2285 | Protease | 0 | 2 / 0Â Â Â Â Â |
| Alcohol dehydrogenase class III | ADH5 | P11766 | CHEMBL4116 | Enzyme | 0 | 6 / 0Â Â Â Â Â |
| Maternal embryonic leucine zipper kinase | MELK | Q14680 | CHEMBL4578 | Kinase | 0 | 4 / 0Â Â Â Â Â |
| Ryanodine receptor 1 | RYR1 | P21817 | CHEMBL1846 | Ligand-gated ion channel | 0 | 1 / 0Â Â Â Â Â |
